# Tiger Salamanders (*Ambystoma tigrinum*) Increase Foot Contact Surface Area on Challenging Substrates During Terrestrial Locomotion

**DOI:** 10.1093/iob/obaa029

**Published:** 2020-09-21

**Authors:** Christine M Vega, Miriam A Ashley-Ross

**Affiliations:** Department of Biology, Wake Forest University, 1834 Wake Forest Road, Winston-Salem, NC 27109, USA

## Abstract

Animals live in heterogeneous environments must navigate in order to forage or capture food, defend territories, and locate mates. These heterogeneous environments have a variety of substrates that differ in their roughness, texture, and other properties, all of which may alter locomotor performance. Despite such natural variation in substrate, many studies on locomotion use noncompliant surfaces that either are unrepresentative of the range of substrates experienced by species or underestimate maximal locomotor capabilities. The goal of this study was to determine the role of forefeet and hindfeet on substrates with different properties during walking in a generalized sprawling tetrapod, the tiger salamander (*Ambystoma tigrinum*). Adult salamanders (*n* = 4, SVL = 11.2–14.6 cm) walked across level dry sand (DS), semi-soft plaster of Paris (PoP), wet sand (WS), and a hard, noncompliant surface (table)—substrates that vary in compliance. Trials were filmed in dorsal and anterior views. Videos were analyzed to determine the number of digits and surface area of each foot in contact with the substrate. The surface area of the forelimbs contacting the substrate was significantly greater on DS and PoP than on WS and the table. The surface area of the hindlimbs contacting the substrate was significantly greater on DS than on all other substrates. There were no significant differences in the time that the fore- or hindfeet were in contact with the substrate as determined by the number of digits. We conclude that salamanders modulate the use of their feet depending on the substrate, particularly on DS which is known to increase the mechanical work and energy expended during locomotion owing to the fluid nature of its loose particles. More studies are needed to test a wider range of substrates and to incorporate behavioral data from field studies to get a better understanding of how salamanders are affected by different substrates in their natural environment.

## Introduction

Locomotion plays an important role in an animal’s ability to obtain food, avoid predation, defend territories, and locate mates. However, because few environments are uniform, animals must be able to move effectively on a variety of substrates. The impact of locomotor performance on fitness has motivated studies of many aspects of locomotion across diverse species. However, many of these studies were performed under laboratory conditions on level, noncompliant surfaces that likely underestimate animal’s ability to make kinematic adjustments that could maximize locomotor performance in natural environments. Quantifying the kinematics of how animals move on noncompliant surfaces is an important first step to understanding animals’ locomotor capabilities, and provides a basis from which to build on the complex interactions between the locomotor strategies of animals and their environment. Collecting data on substrates that better match those encountered in nature can also provide useful insight into the behavioral plasticity of animals.

It is also important to consider how kinematics might change when an animal comes across changes in slope, substrate texture, or other properties. Many previous studies in a range of taxa (e.g., primates, marsupials, rodents, lizards, and snakes) have investigated the effects of substrate on locomotor performance (Kelley et al. 1997; Irschick and Jayne 1998; Irschick and Losos 1999; Kohlsdorf et al. 2001). A diversity of substrate definitions have been used in previous studies such as branch diameter (Lammers and Biknevicius 2004; Shapiro and Young 2010), incline (Irschick and Jayne 1998), and perch choice (Irschick and Losos 1999; Mattingly and Jayne 2005; Mansfield and Jayne 2011; Hyams et al. 2012; Jones and Jayne 2012). The ability to grip the surface of a substrate is a key feature for locomotion across species and environments, and even taxa with different body shapes have converged on similar strategies (Cartmill 1985). For example, arboreal snake species like boa constrictors and rat snakes rely on friction to grip the branch surface, which is similar to the strategy used by primates and opossums with prehensile tails (Cartmill 1985; Lammers and Biknevicius 2004).

Substrate roughness changes the surface area available for animals to grip during locomotion and in many instances it improves grip during locomotion (Brandt et al. 2015). Substrate roughness can improve the force exchange during stance phase to generate forward thrust (Höfling et al. 2012). However, the effects of substrate roughness and how that influences grip during locomotion in level terrestrial environments are less understood. Locomotion requires an interaction between the animal’s anatomy (i.e., the feet, limb lengths, and muscle force production) and characteristics of the substrate such as incline and friction.

Previous studies on substrate focused on the effects between habitat structure and a resulting decrease in lizard locomotor performance (decreased speed) on inclined surfaces (Irschick and Jayne 1998, 1999). There have been studies on how locomotion on different substrates affects running speed (Kohlsdorf et al. 2004; Vanhooydonck et al. 2005, 2015), but these effects are less understood and results are mixed. Variation in substrate affected some types of locomotor performance (speed, acceleration, and stamina) in lacertid lizards, but not consistently; the effects were species-dependent for measures of speed and stamina, but not for acceleration (Vanhooydonck et al. 2015). There is also some evidence that habitat distribution between species is not correlated to locomotor performance. Sprinting ability in a broadly distributed lizard species (*Callisarus draconoides*) and a dune-dwelling species (*U. scoparia*) was similar on fine and coarse sand (Korff and McHenry 2011). Phenotypes evolved under multiple selective pressures and are likely not strongly optimized or directly selective for locomotion on particular substrates (Garland and Losos 1994; Kelley et al. 1997; Irschick and Jayne 1998; Kohlsdorf et al. 2001). Other studies on the effects of locomotion on different substrates often focus on morphological adaptations for specific substrates (Carothers 1986) or on comparing specialists and generalists to determine phylogenetic effects (TulLi et al. 2012). The relationships among locomotor strategy, performance, and habitat structure are complex and patterns may also be influences by the substrates animals that are moving on in their natural environments. Most of the previous studies use whole-body measurements to assess the effects of substrate, but there are not many studies that have investigated what the feet themselves are doing in response to substrate variation (Li et al. 2012).

Whole body measurements are useful ways to assess performance and fitness, but the feet might play an important role in animals’ abilities to handle substrate variation because the foot is the part of the animal that contacts and interacts with the substrate. Environmental structure’s effect on locomotor performance is a classical assumption of Arnold’s paradigm (Arnold 1983) and evidence supporting this assumption is common among squamates (Garland and Losos 1994) Although several squamate species have locomotor adaptations to habitat structures (Garland and Losos 1994), there is also evidence that generalist lizards show variation in maximum sprint speeds on different substrates due to friction (Brandt et al. 2015). Tiger salamanders (*Ambystoma tigrinum*) are an interesting model system to use because they have a generalized sprawling-postured body plan with no specialized morphological adaptations for locomotion on particular substrates. Animals’ may actively control the surface area of their feet in contact with the substrate in response to differences in compliance or deformability in response to body weight. There is evidence from previous studies of climbing and terrestrial locomotion that locomotor performance can be affected by compliance (Ferris et al. 1999; Byrnes and Jayne 2010; Spence et al. 2010; Channon et al. 2011). Animals use various strategies to conform to shapes in their environment to effectively apply gripping and propulsive forces during locomotion depending on their body plan and behaviors (Byrnes and Jayne 2010). The substrate can conform to the shape of the animal’s body that is in contact or the animal can actively control its body and conform to the shape of the substrate (Byrnes and Jayne 2010). Substrate compliance presents difficulties to stability and energy (Bonser 1999), behavioral adjustments to the mode of locomotion (Thorpe et al. 2009; Byrnes and Jayne 2010), and kinetic dynamics (McMahon and Greene 1979; Byrnes et al. 2008) all of which may have surprising costs to locomotion (Gilman et al. 2012). Although the effects of substrate compliance in the context of climbing, jumping, and leaping in arboreal habitats have been well documented, the effects of substrate compliance in other terrestrial contexts, like walking, are less understood. However, the challenges of walking and running on sand have been studied and can offer some insight.

The effort to move on soft, yielding substrates is greater than the effort required for moving across a hard, level surface. Locomotion on compliant substrates, like dry sand (DS), increases both the mechanic work, energy expended, and gait stability (Lejeune et al. 1998; Kerdok et al. 2002). DS is unstable as the sand particles are free to move over each other; therefore, salamanders should increase foot contact with this substrate to maintain balance during locomotion. Traditionally, forelimbs were thought to produce most of the braking ground reaction force (GRF) while hindlimbs produce most of the accelerative GRF (McElroy et al. 2014), but this does not seem to be the case for all species so it is useful to separate the limbs during analysis. Despite the unequal sizes of salamander limbs, both limbs experience similar mediolateral and vertical components of GRF which suggests that fore and hindlimbs have comparable contributions to body support (Kawano and Blob 2013). Animals use substrate-specific locomotion strategies because the limbs of animals show different morphology and kinematics adapted for their environment. For example, the desert-dwelling zebra-tailed lizard (*C. draconoides*) switches between digitigrade and plantigrade foot postures depending on whether it is running on a solid or granular surface (Li et al. 2012). The switch between these foot postures may also have implications for how much of the foot contact the substrate.

The goal of this study was to determine if salamanders adjust how they use their feet on substrates with different physical properties. Salamanders do not have any morphological adaptations for particular substrates and may be able to adjust how their foot interactions with the substrate to limit performance consequences. Generalists are known to behaviorally modify their kinematics to maximize performance on different substrates (Brandt et al. 2015). Salamanders were filmed walking across the following substrates: DS, plaster of Paris (PoP; simulates fine-grained mud), wet sand (WS), and a hard, noncompliant surface (HNC), a table. These surfaces all varied in their compliance (deformability in response to body weight) and when listed will always be ordered from most to least compliant for ease of interpretation. The number of digits and projected surface area of each individual foot in contact with the substrate was quantified. These measures were used to calculate integrated surface area of the forelimbs and hindlimbs in contact with the substrate throughout each complete stride cycle, and the percent stride where a foot was considered in contact with the substrate. We tested two hypotheses: (1) We predicted that salamanders would make kinematic adjustments on the more challenging substrate leading to greater foot surface area in contact with the substrate (DS and PoP) compared with the other substrates tested (WS and HNC). (2) Salamanders should also leave each digit in contact with the substrate for a greater portion of the stride cycle in locomotion on the DS substrate.

## Methods

### Experimental animals

Wild-caught tiger salamanders (SVL = 11.2–14.6 cm) were donated by the laboratory of Jacob Kerby at the University of South Dakota (Vermillion, SD). Salamanders were housed individually in aquaria with access to a terrestrial landing and kept on a 12:12 light: dark cycle. All experimental trials were performed with four metamorphosed, adult individuals. All procedures were approved by the [redacted for review] University IACUC (protocol A16-171).

### Filming set-up and data collection

Tiger salamanders (*n* = 4) were filmed with two PHANTOM version 310 cameras (Vision Research, Wayne, NJ) at 100 fps in a dorsal and anterior view. All measurements were taken from the dorsal view, but the additional in-plane view provided another vantage point to view the feet and assist with video analysis. Cameras were synchronized using a common trigger system. Substrates were each put into 0.7 × 1.0 m aluminum trays; there was an individual tray for every trial. After the substrate filled the tray’s depth, the substrate was evenly distributed and leveled. A 1 × 1 cm grid was visible on the tray for scaling purposes. All four individuals were filmed on each of the following substrates: DS, PoP, WS, and an HNC. Quikrete Premium Play sand and DAP PoP were purchased locally. WS was at its water-holding capacity. PoP was mixed (2:1 dry mix to water ratio) and allowed to set until its consistency was comparable to moist mud before trials began. Animals selected their own speeds for all trials.

To begin a trial, a tray was placed within the filming arena and a salamander was positioned in the tray. The base of the tail was gently squeezed to prompt initial movement and salamanders were squirted with water from a spray bottle to encourage continuous, steady locomotion across the middle of the tray. Trials during which the salamander maintained steady locomotion and crossed the entire distance of the tray were used for subsequent analysis. If a trial was deemed unusable, the substrate was re-distributed and smoothed before a new trial began for DS and WS. If a PoP trial was deemed unusable, then a new PoP mixture was made to control for drying over the course of trials. If an animal had >3 false starts, then a new animal was used, so the previous animal could rest before more attempts were tried. The rest period for the previously used animal was at least 30 min.

Videos (*n* = 16) were analyzed frame-by-frame using ImageJ software (National Institute of Health, Bethesda, MD, USA) and were subsampled to 30 fps. The ability of salamanders to make kinematic adjustments based on substrate type was quantified by measuring the projected surface area of each foot and number of digits in contact with each substrate. The absolute surface area of the foot is roughly constant throughout the experiment because it is a function of the salamander’s foot size. However, the contact surface area between the foot and substrate will vary during locomotion because (1) compliant substrates can conform to the relatively rigid foot and (2) the compliant foot can also conform to the relatively rigid substrates. A projected surface area of the foot on the substrate from a dorsal view was measured. The projected surface area of each individual foot was measured frame-by-frame in the video using the freehand selections tool. Projected surface area measurements included any digits and palm (up to the wrist joint) that were in contact with the substrate for a given frame. Across all frames in a stride, the projected surface area in contact (measured in cm^2^) was integrated across the percent stride (%) yielding units of %*cm^2^ which standardizes for trials of different absolute durations. Here, we used the algebraic equivalent to the integration which takes the summation of the areas created between each measurement point. These data were used to calculate the integrated surface area of the forelimbs and hindlimbs that was in contact with the substrate. Counts of the number of digits in contact with the substrate were also recorded frame-by-frame to calculate the percentage of the stride that each individual foot was in contact with the substrate. The numbers of digits chosen as the threshold for a foot to be touching were selected so that at least 50% of the digits on the forefoot or hindfoot were in contact with the substrate (i.e., two of the four digits on the forefeet, and three of the five digits on the hindfeet). The subset of frames in each stride that had a digit count >50% threshold was used to determine for what percentage of that particular stride each foot was in contact.

### Statistical analysis

To compare locomotor trials, the duration of each cycle was converted to a percentage since not all cycles were of the same absolute duration. Integrated surface area and percentage of stride touching calculations were both compared over multiple strides (*n* = 82; Table 1) from four individuals. All calculations and statistical analyses were performed in R (version 3.4.3).

**Table 1:** Stride sample size by substrate and individual

Treatment	Number of strides by individual			
	G	I	K	L
DS	3	3	3	5
Plaster of Paris	6	7	4	6
WS	5	7	2	4
Hard, noncompliant	9	7	6	6

One video per salamander per treatment was analyzed, but there were multiple strides were collected in each video.

We used a general linear mixed model to test for differences in the integrated surface area contacting the substrate and percent stride touching the substrate among forelimbs and the hindlimbs across treatment groups. Individual was included as a random effect. *Post hoc* differences were assessed using Tukey’s  hHonestly Significant Difference Snout-vent length, with the Holm–Bonferroni correction for multiple comparisons. All data were log-transformed prior to analysis, and significance was assessed at *α* = 0.05 level. For ease of interpretation, non-transformed data are represented in the figures.

## Data availability statement

The data that support the findings of this study are available from the corresponding author upon request. Authors are responsible for data archiving. To promote the preservation and fuller use of data, Integrative Organismal Biology actively encourages data archiving in appropriate public data libraries.

## Results

Salamanders selected their own speeds for each locomotor trial. Data are reported as means ± standard error. Salamanders selected slightly slower speeds on DS (0.019 ± 0.005 m/s) compared with the other substrates. Salamanders selected similar speeds on PoP (0.024 ± 0.003 m/s) and HNC (0.021 ± 0.002 m/s) and faster speeds on WS (0.031 ± 0.006 m/s). Stride lengths were standardized by SVL . Salamanders had greater standardized stride lengths on DS (14.32 ± 0.70 m/m) compared with PoP (13.16 ± 0.69 m/m), WS (11.31 ± 0.68 m/m), and HNC (7.88 ± 0.24 m/m). Representative walking sequences across each substrate are presented in Supplementary data, Videos S1–S4.

Salamanders responded to changes in substrate compliance by increasing the surface area of their forelimbs and hindlimbs in contact with the substrate (Tables 2 and 3). This response was substrate-dependent. In contrast, substrates differed little in the percentage of the stride cycle for which the forefeet and hindfeet were in contact (Tables 4 and 5).

**Table 2:** Mean integrated surface areas (%*cm^2^) and standard errors for the forefeet and hindfeet for each substrate treatment

	Forefeet	Hindfeet
	Mean ± SE	Mean ± SE
DS	520 ± 27	943 ± 69
Plaster of Paris	510 ± 23	784 ± 50
WS	429 ± 30	743 ± 43
HNC	413 ± 20	772 ± 36

Mean values ± standard error of the mean integrated surface area for tiger salamanders (*n* = 4 individuals). Surface area was measured for the part of each foot up to the wrist joint in contact with the substrate frame-by-frame. The integration of the surface areas for the forefeet and hindfeet were each calculated.

**Table 3:** Results of a general linear mixed model performed on mean integrated surface area values for the forefeet and hindfeet on substrates of varying compliance

	Forefeet	Hindfeet
Model	**2.32** **×** **10^−11^**	**0.001502**
DS vs. HNC	**4.05** **×** **10^−07^**	**0.003771**
WS vs. HNC	0.171964	1
Plaster of Paris vs. HNC	**2.24** **×** **10^−09^**	1
WS vs. DS	**0.001517**	**0.004489**
Plaster of Paris vs. DS	0.996093	**0.002681**
Plaster of Paris vs. WS	**0.000278**	1

A general linear mixed model with individual as a random effect was performed (*α* = 0.05). *P* values are reported for the model, as well as pairwise *post hoc* comparisons. Bold indicates significance.

**Table 4:** Mean percentage of the stride the forefeet and hindfeet are contacting substrates of varying compliance

	Forefeet	Hindfeet
	Mean ± SE	Mean ± SE
DS	50.56 ± 4.04	64.50 ± 3.90
Plaster of Paris	58.66 ± 2.63	60.38 ± 4.23
WS	54.71 ± 2.69	62.40 ± 2.72
HNC	50.66 ± 1.57	51.12 ± 3.63

Mean values ± standard error of the mean integrated surface area for tiger salamanders (*n* = 4 individuals). A foot was considered in contact with the substrate if at least 50% of the digits were touching.

**Table 5:** Results of a general linear mixed model performed on mean percentage of the stride the forelimbs and hindlimbs are contacting substrates of varying compliance

	Forefeet	Hindfeet
Model	**0.036012**	**0.037847**
DS vs. HNC	0.731207	0.096113
WS vs. HNC	0.731207	0.088690
Plaster of Paris vs. HNC	0.103108	0.391323
WS vs. DS	0.471228	1
Plaster of Paris vs. DS	0.066237	1
Plaster of Paris vs. WS	0.731207	1

A general linear mixed model with individual as a random effect was performed (*α* = 0.05). *P*-values are reported for the model, as well as pairwise *post-hoc* comparisons. Bold indicates significance.

**Fig. 1:**
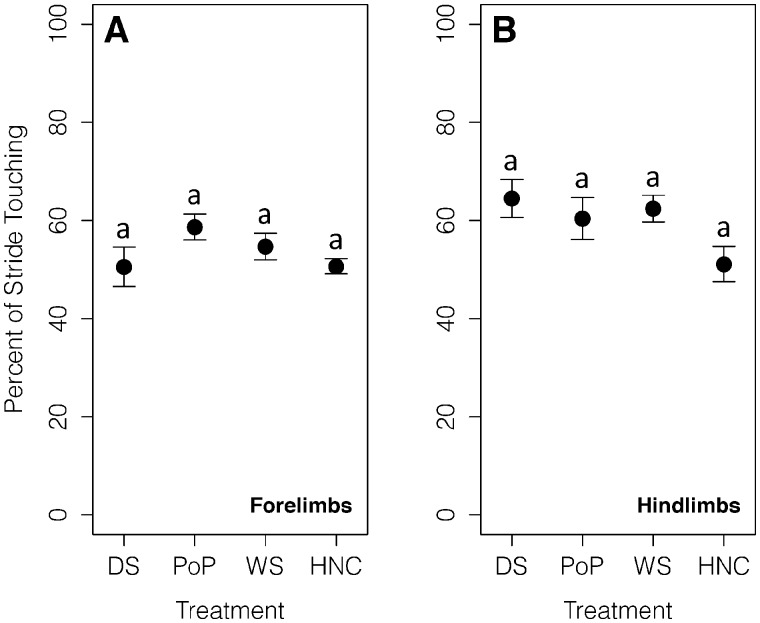
Integrated surface area (%*cm^2^) for the forelimbs and hindlimbs in contact with a DS, PoP, WS, and an HNC and during walking. Points are means of complete stride cycles (*n* = 82) from four individuals. Error bars are standard errors of the mean. Substrates are ordered from most to least compliant. (**A**) The integrated surface area of the forelimbs on DS and PoP was significantly greater than on HNC and WS. There were no significant differences in integrated surface area of the forelimbs between HNC and WS or PoP and DS. (**B**) The integrated surface area of the hindlimbs on DS was significantly greater than on HNC, WS, and PoP. There were no significant differences in integrated surface area of the hindlimbs between HNC and WS, PoP and HNC, or PoP and WS.

The integrated surface area of the forelimbs on DS was significantly greater than on HNC and WS (*P*⋘0.05; Fig. 1A). The integrated surface area of the forelimbs on PoP was significantly greater than on HNC and WS (*P*⋘0.05; Fig. 1A). There were no significant differences in integrated surface area of the forelimbs between HNC and WS or PoP and DS (*P* > 0.05; Fig. 1A).

The integrated surface area for the hindlimbs on DS was significantly higher than on HNC, WS, and PoP (*P* ≪ 0.05, respectively; Fig. 1B). There were no significant differences in integrated surface area of the hindlimbs between HNC and WS, PoP and HNC, or PoP and WS (*P* > 0.05; Fig. 1B).

We considered the potential confounding effects associated with different body sizes (and, thus, potentially different foot surface areas among individuals). In a subsequent analysis (data not shown), we standardized the surface area of each foot by the maximum size of each foot for each individual. We performed the same statistical analysis on these standardized integrated surface area data and the results were not different from the raw surface area values presented here.

**Fig. 2:**
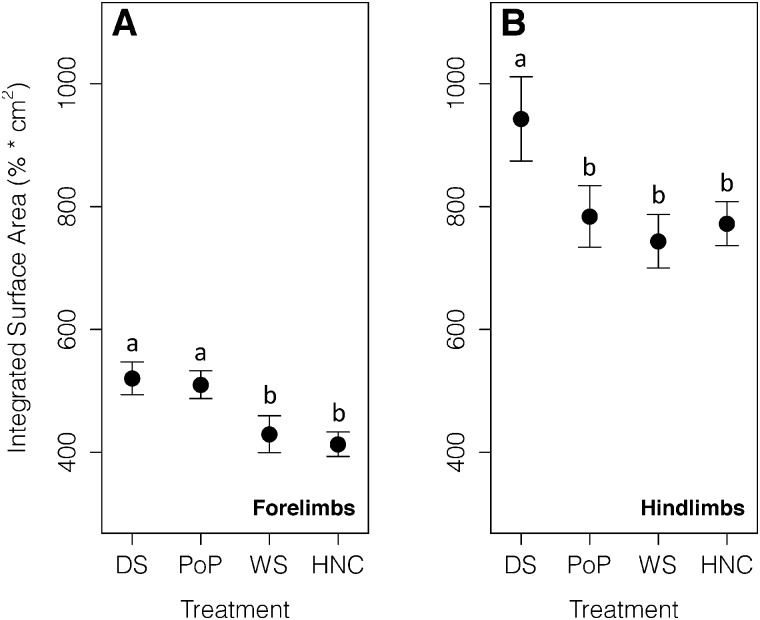
Percent stride of the forelimbs and hindlimbs in contact with DS, PoP, WS, and an HNC during walking. Points are means of complete stride cycles (*n* = 82) from four individuals. Error bars are standard errors of the mean. Substrates are ordered from most to least compliant. (**A**) There were no significant differences in percent stride touching among any of the substrates for the forelimbs. (**B**) There were no significant differences in percent stride touching among the substrates for the hindlimbs.

Although the models were significant (*P* < 0.05; Table 4), none of the pairwise *post hoc* comparisons were significant for both the forefeet and hindfeet (*P* > 0.05; Fig. 2A and B; Tables 3 and 4).

## Discussion

We predicted that salamanders would adjust the area and duration of the stride cycle that their feet were in contact with the substrate based on substrate type and properties. Our results provided support for the hypotheses tested. Salamander forelimbs had more surface area in contact with the substrate when walking on DS and PoP, whereas salamander hindlimbs had more surface area in contact with the substrate when walking on DS in comparison to locomotion on other substrates. There was a performance consequence of slower speed on DS, but the increased standardized stride length may have helped to mitigate that consequence. Overall, there were no significant differences in the duration of the percent stride cycle that the forefeet and hindfeet were in contact for any substrate.

Some studies have examined how soft, yielding substrates alter the mechanics and energetics of locomotion compared with noncompliant substrates in order to understand how kinematics are affected by substrate properties (Lejeune et al. 1998; Kerdok et al. 2002). Salamander forelimbs and hindlimbs had more surface area in contact with the DS compared to the other substrates likely due to the increased energetic demands and loosely packed sand particles. The effects of substrate properties on mechanics and energetics are well documented in humans; our results generally support those of previous studies. Walking on sand requires 1.6–2.5 times more mechanical work than walking on a noncompliant, hard surface and 2.1–2.7 times more energy (Lejeune et al. 1998). The increase in mechanical work for walking is due primarily to increases in the muscle–tendon work, as well as work done on the sand by the foot (Lejeune et al. 1998). Studies have shown that the energy required for locomotion on sand increases because energy is lost to the substrate when the surface is deformed (Li et al. 2012) and drag increases when the feet penetrate the substrate during locomotion (Claussen et al. 2002). Although we did not quantify mechanical work or energy expenditure, it seems reasonable that, in salamanders, the surface area of their forelimbs and hindlimbs in contact with DS during locomotion might increase for similar reasons. Locomotion on compliant substrates may also result in changes in other parameters such as leg stiffness (Kerdok et al. 2002), speed (Herreid and Full 1986), and stride length (Claussen et al. 2002). Although salamanders’ increased their standardized stride length on DS, the decrease in speed may have been due to a decreased ability to grip the sand substrate. A generalist lizard ran slowly on thin and coarse sand and these substrates had low grip values (Brandt et al. 2015).

As was the case with DS, salamander forelimbs also had greater surface area in contact with PoP. Very few previous studies have used substrates comparable to PoP (a surrogate for fine-grained mud). Previous studies on gecko adhesive ability during locomotion on wet substrates found that neither water nor substrate type (glass vs. acrylic) had a significant effect on sprint velocity (Stark et al. 2015; Garner et al. 2017). It is difficult to make direct comparisons because these studies sprayed water on the surface of substrates incapable of holding water. These gecko adhesion studies, however, emphasize that animals can make kinematic and behavioral adjustments which allow them to handle substrate variations in their natural environment. There were similar responses in sprint performance to wet substrates, but variation in stopping behavior, slipping, and toe pad wetting may be explained by habitat differences (Stark et al. 2015). Note that the salamanders in our study walked at slow speeds over relatively short distances, so it is also possible that substrate type may play a more consistent role over longer distances and in circumstances under which salamanders reach maximal locomotor performance. Although the PoP particles would not have behaved similarly to loose sand, we speculate that the salamanders increased surface area contact of the forelimbs to prevent slipping and maintain balance as forelimbs play a role in braking (McElroy et al. 2014).

The probability of an animal slipping during locomotion depends on the substrate’s frictional properties (van der Tol et al. 2005). When friction between the substrate surface and the feet is too low, slips can occur and it is this interaction between the foot and substrate that determines the friction coefficient (Alexander 2003). Frictional forces are especially important for speed because the foot needs to exert force to the substrate when the animal steps during locomotion(Lejeune et al. 1998; Kerdok et al. 2002; Korff and McHenry 2011). It is interesting that there was no significant difference in speed on PoP and HNC. The differences observed in the forelimbs are therefore unrelated to speed, and must be substrate dependent.

Many components of habitat structure in a salamander’s natural environment, such as leaf litter, fallen logs, and rocks, can provide opportunities for salamanders to hide from predators. The complexity of salamander habitat structure may limit the distance these animals must typically move to find cover. Salamanders may depend on other strategies for predator avoidance, using locomotion as a last resort. Predators that rely on color vision may differentially target color morphs; this is a common explanation for the evolution and maintenance of polymorphisms in populations (Stimson and Berman 1990). Two color morphs of the eastern red-backed salamander (*Plethodon cinereus*) exhibit different antipredator behaviors and are targeted by predators at different rates (Venesky and Anthony 2007). Additionally, there is some evidence for size-mediated predation pressure for salamander communities in the southern Appalachians which are known for their salamander diversity. These salamanders are morphologically similar, but show differences in size and habitat; larger species are found in aquatic habitats and smaller species are found in terrestrial habitats (Hairston 1949). It is possible that small salamanders avoid microhabitats with predators, whereas larger salamanders coexist with large predators (Formanowicz and Brod 1993) perhaps relying on tail autotomy as an antipredator strategy. Previous studies indicate that there are a range of behaviors available that might explain why our salamanders did not demonstrate significantly different contact time with each substrate.

When an animal is moving on a soft, yielding substrate, its locomotor performance may suffer due to the low penetration resistance of the substrate. A previous study investigating the role of appendage design in locomotion on flowable substrates found that animals actively adjust their gait frequency to maintain effective locomotion; however, gait frequency modulation was not enough to explain changes in speed suggesting there may be passive control mechanisms (i.e., within-stride kinematic variations) (Qian et al. 2015). It is likely that salamanders combine passive and active control on challenging substrates such as DS which permits maintenance of performance despite changes in substrate stiffness. Animals can use different strategies to conform to shapes in their environment to assist with gripping and propulsion during locomotion (Byrnes and Jayne 2010). The substrate can passively conform to the shape of the foot or the animal can actively control its foot shape to conform to the substrate. For example, DS coming into increased contact with the foot because it is a soft, yielding, and conformable substrate is passive control. Conversely, muscle control of foot shape and interaction with the substrate is active control. It is also possible that the increased surface area results seen in this study are the passive result of the foot going into the yielding substrate more and not an active control mechanism.

While the salamanders in our study did increase the surface area in contact with flowable substrates, they did not also adjust the time the forefeet or hindfeet were in contact with any substrate. It is yet unclear whether these patterns are a result of active control by the animal, or a passive consequence of substrate compliance. Our study reinforces the need to study how animals adjust their locomotion on substrates that vary in their properties (i.e., roughness, wetness, compliance, etc.) as models for the conditions that animals may encounter in their natural environment. Further investigations are needed to determine how changes in substrate affect locomotor performance in the context of species, morphology, and ecology.

## Supplementary Material

obaa029_Supplementary_Data
